# Editorial: Methods for Synaptic Interrogation

**DOI:** 10.3389/fnsyn.2020.00023

**Published:** 2020-06-10

**Authors:** Dirk Feldmeyer, John F. Wesseling, P. Jesper Sjöström

**Affiliations:** ^1^Institute of Neuroscience and Medicine 10 (INM-10), Research Centre Jülich, Jülich, Germany; ^2^Department of Psychiatry, Psychotherapy and Psychosomatics, Medical School, RWTH Aachen University Hospital, Aachen, Germany; ^3^Instituto de Neurociencias, CSIC-UMH, San Juan de Alicante, Spain; ^4^Centre for Research in Neuroscience, Brain Repair and Integrative Neuroscience Program, Department of Medicine, Department of Neurology and Neurosurgery, The Research Institute of the McGill University Health Centre, Montreal General Hospital, Montreal, QC, Canada

**Keywords:** synapse, plasticity, electrophysiology, microscopy, optogenetics, methods

## Introduction

Synapses are the specialized junctions that transmit information between neurons and that connect them into circuits. Synapses are often plastic and can be modulated to process information in the short term as well as to store information in the long term (Zucker and Regehr, 2002; Abbott and Regehr, 2004; Markram et al., 2012; Maheux et al., 2016). They play fundamental roles in biological computation and memory formation—key tasks of the brain. Yet precisely how this occurs remains unknown at levels ranging from molecules to systems.

A wealth of powerful techniques for interrogating synaptic function has emerged in recent years, and yet important studies continue to additionally rely on methods in use decades ago. Training new researchers in the state of the art is a time-consuming bottleneck that is often hampered by the lack of easy-to-understand descriptions of best practices. Here we provide a collection of straightforward, accessible papers on a range of methods for exploring synaptic function, written by experts.

## Methods Described in this Collection

### Finding Synaptic Connections

To analyze synaptic transmission, it is paramount to use clearly identified synaptic connections. Deep insight has previously been obtained by studying classic preparations such as neuromuscular junctions of many species, the Mauthner cell in fish, and the calyx of Held in the mammalian brain stem (e.g., Del Castillo and Katz, 1954; Korn and Faber, 2005; Neher, 2017). In this collection, four articles describe how new neuronal connections can be identified and investigated at the levels of structure and function.

Cultured neurons forming autaptic synapses, i.e., those established by a neuron onto itself, provide an elegant tool allowing the study of synaptic transmission under tightly controlled conditions. Bekkers describes the procedures to prepare and record from autaptic cultures as well as several applications, such as the study of vesicular release, the role of synaptic proteins, and the neuromodulation of synaptic release.

Qi et al. review several applications of the *in-vitro* slice preparation to investigate structure-function aspects of synaptic transmission at connections between identified neurons, e.g., synaptic efficacy and release probability, as well as the location of synaptic contacts. Due to ease of drug application compared to *in-vivo* studies, acute slices are also useful to study neuromodulation of neurotransmitter release. It is also the method of choice to study synaptic transmission in human brain tissue.

Jouhanneau and Poulet describe sophisticated experimental procedures they developed to study monosynaptically connected neurons in superficial layers of intact rodent brain. Using two-photon microscopy for multiple targeted patching, they are able to characterize how excitatory and inhibitory neuronal microcircuits act *in vivo*, under realistic physiological conditions and behavioral states. The use of multiple electrodes additionally permits the study of connectivity profiles in intact neuronal microcircuits.

Besides local connections, identification of long-range monosynaptic connections is an important step to unravel paths across brain areas. In their original paper, Lavin et al. report procedures for labeling monosynaptic connections of a specific neuron population using modified rabies virus injection in combination with a preceding injection of helper viruses (also see Wickersham et al., 2007). They found that the helper virus concentration was a key determinant for the quality of tracing results.

### Exploring Plasticity and Synaptic Release

Long-term potentiation and depression are widely thought to underlie learning and memory, yet the locus of plasticity expression is still a matter of debate (Costa et al., 2017). Four articles in this Research Topic describe a range of electrophysiological and optical imaging techniques and analytic frameworks that enable the elucidation of the locus of expression. Glasgow et al. review the strengths and pitfalls of a range of methods, including classical tools such as spontaneous release, paired-pulse ratio, and NMDA:AMPA ratio, to more novel optical tools including calcium indicators and optical actuators. Brock et al. delve deeper into CV analysis, providing a detailed but straightforward how-to guide to this classical method, including derivation of formulas, computer simulations, and potential pitfalls. Although paired recordings may alleviate some of the pitfalls, even monosynaptic inputs typically arise at multiple sites, which can complicate the analysis. However, in two complementary methods papers, MacDougall and Fine and Padamsey et al. show how key complications can be avoided by combining electrophysiology with two-photon microscopy of transmission at single sites in acute slices.

A better understanding of short-term plasticity is needed for understanding how biological computation works, but remains a complicated effort. Bykowska et al. review new analytic procedures for using underlying principles to help infer both release statistics and synaptic dynamics from data ranging from unitary connections to *in-vivo* network recordings. However, a complication is that the underlying principles have never been completely resolved.

For example, many models assume that individual synapses can only release transmitter from a single vesicle at a time. However, a seemingly contradictory concept that is also widespread is that individual synapses maintain a readily releasable pool of multiple synaptic vesicles. And indeed, Barros-Zulaica et al. present a detailed analysis of monosynaptic connections indicating that at least some types of synapses can and often do release transmitter from multiple vesicles within this pool simultaneously, in agreement with several other studies (Rudolph et al., 2015). Soares et al. reach a similar conclusion from a complementary study using the glutamate detector iGluSnfr to circumvent caveats associated with electrophysiological and less direct optical imaging techniques. Soares et al. also introduce a new method to eliminate measurement biases related to failures of neurotransmission that may be adaptable to a broad range of techniques.

Furthermore, even models that allow for multivesicular release often assume uniform release probability among the vesicles within the readily releasable pool. However, Gustafsson et al. review their own early evidence from neonatal hippocampus that many vesicles typically categorized as readily releasable only undergo exocytosis after persistent repetitive stimulation. The phenomenon is likely related to the current concept of reluctant or slowly releasing subdivisions of readily releasable pools, now reported at a wide range of synapse types. By themselves, the results do not necessarily contradict assumptions about uniform release probability because the delayed exocytosis may involve post-primed vesicles that were not actually readily releasable at the beginning of stimulation. Nevertheless, as part of a review of complications that can interfere with measurements of vesicle recruitment to the readily releasable pool, Wesseling summarizes evidence from calyx of Held that seems incompatible (also see Maschi and Klyachko, 2020).

### Two-Photon Neurotransmitter Uncaging

Neurotransmitter uncaging can act within milliseconds, making it a powerful method for elucidating synaptic function, but with traditional light sources such UV lamps or violet lasers, uncaging is not spatially confined. With two-photon excitation, however, the uncaged volume is smaller than a μm^3^, providing biological realism and excellent experimental control.

Ellis-Davies—a pioneer of uncaging—describes the state of the art of two-photon glutamate uncaging. He compares the pros and cons of using different cages and provides historical background. Building on this work, the Araya team describes in detail a cost-effective custom-built microscope for cutting-edge combined two-photon uncaging and imaging (Mitchell et al.). Relying on a single ultrafast laser to save cost, this cutting-edge setup can still activate multiple dendritic spines with near simultaneous calcium imaging.

### Combining Genetics and Optics

Optogenetics is a powerful technique for interrogating neural circuits, yet its implementation is not always straightforward. In a practical how-to paper, Gruver and Watt show how to optimize optogenetic activation of cerebellar Purkinje cell axons to interrogate inputs to the deep cerebellar nuclei. The concept of optogenetics is often extended to include the monitoring of cellular compartments, e.g., using genetically encoded calcium indicators. Brockhaus et al. coupled GCaMP6f to synaptophysin to create synGCaMP6f, which enriches the calcium indicator in boutons, resulting in excellent signal to noise in small axonal compartments.

It is also possible to combine optical and genetic methods to explore synaptic architecture. Reshetniak and Rizzoli provide a detailed review of approaches for visualizing synaptic organelles and cytoskeletal protein complexes, such as styryl dyes, cypHer5E-labeled antibodies, quantum dots, toxins, etc. Finally, Ebner et al. provide a detailed protocol for how to optically induce calcium-dependent gene activation and labeling of active neurons using CaMPARI and Cal-Light. Because CaMPARI acts in seconds but Cal-Light requires several days, these powerful tools can serve different purposes on different timescales.

## Concluding Remarks

This collection of papers provides the current state of a sampling of useful methods for interrogating synaptic function. We gathered these papers to make accessible a range of techniques, by disseminating a set of how-to descriptions written by the experts themselves. Our hope is that this collection will serve as a useful foundation for future synaptic neuroscience research, and a new generation of important discoveries about the nature of brain function.

## Author Contributions

DF, JW, and PS wrote the manuscript.

## Conflict of Interest

The authors declare that the research was conducted in the absence of any commercial or financial relationships that could be construed as a potential conflict of interest.
